# Treatment of Dermatological Conditions Associated with HIV/AIDS: The Scarcity of Guidance on a Global Scale

**DOI:** 10.1155/2016/3272483

**Published:** 2016-05-03

**Authors:** Suchismita Paul, Rachel Evans, Toby Maurer, Lulu M. Muhe, Esther E. Freeman

**Affiliations:** ^1^Harvard Medical School, Boston, MA, USA; ^2^HIV Department, World Health Organization, Geneva, Switzerland; ^3^Department of Dermatology, University of California San Francisco School of Medicine, San Francisco, CA, USA; ^4^Department of Maternal, Child and Adolescent Health, World Health Organization, Geneva, Switzerland; ^5^Department of Dermatology, Massachusetts General Hospital, Harvard Medical School, Bartlett Hall 6R, 55 Fruit Street, Boston, MA 02114, USA

## Abstract

*Background*. Skin diseases associated with Human Immunodeficiency Virus (HIV) infection are associated with significant morbidity and mortality. In resource-limited settings, nondermatologists and lay health care providers on the front line of HIV care provide much of the treatment for these conditions.* Objective*. To evaluate guidelines for treatment of HIV-related skin conditions and assess their accessibility, comprehensiveness, and quality of evidence employed.* Methods*. A review was undertaken of all national and society guidelines which included treatment information on the ten highest burden HIV-related skin conditions. The search strategy included gray and peer-reviewed literature.* Results*. Of 430 potential guidelines, 86 met inclusion criteria, and only 2 were written specifically to address HIV-related skin diseases as a whole. Treatment information for HIV-related skin conditions was embedded within guidelines written for other purposes, primarily HIV/AIDs treatment guidelines (49%). Development of guidelines relied either partially or completely on expert opinion (62%). Only 16% of guidelines used gradation of evidence quality and these were primarily from high-income countries (*p* = 0.001).* Limitations*. Due to the nature of gray literature, not all guidelines may have been identified.* Conclusion*. This review highlights the need for evidence-based summary guidelines that address treatment for HIV-related skin conditions in an accessible format.

## 1. Introduction

In 2013, the number of people living with Human Immunodeficiency Virus (HIV) was estimated to be 35 million (31.8 million adults and 3.2 million children < 15 years) globally [1]. There were 2.1 million new HIV infections (1.9 million adults and 240,000 children) and 1.5 million deaths due to HIV-related causes (1.3 million adults and 190,000 children) [1]. The major causes of mortality are Acquired Immunodeficiency Syndrome (AIDS) related mortality or from opportunistic infections such as tuberculosis (TB) and cryptococcal infections. The initiation of combination antiretroviral therapy (cART) has led to a profound impact on mortality, averting 7.6 million deaths globally since 1995 [1].

Globally, skin conditions are the fourth leading cause of nonfatal disease burden in terms of years lost due to disability, ahead of conditions such as diabetes and COPD [2]. HIV-related skin conditions are important in terms of burden, impact on quality of life, and associated mortality. Prior to availability of effective cART, it was estimated that up to 90% of HIV-infected individuals have associated skin and mucosal conditions during the course of their illness [3]. Skin manifestations may be the first sign of HIV infection and therefore present an opportunity for HIV testing and earlier diagnosis [3]. Some mucocutaneous conditions in particular are a proxy indicator for more advanced immunodeficiency and the need for prompt initiation of cART [4, 5]. Certain skin conditions can also lead to severe morbidity such as pain on swallowing from oropharyngeal candidiasis and recurrent infections that are difficult to treat, such as scabies [6, 7]. Other conditions such as zoster and extensive tinea that are difficult to conceal may cause stigma and cause psychosocial stress and depression [8].

Particular challenges in the effective management of HIV-related skin conditions include the fact that they are difficult to treat and may recur more frequently compared to immunocompetent individuals in the absence of immune reconstitution with cART [9, 10]. Early recognition of HIV-related skin conditions presents the opportunity for earlier HIV diagnosis and cART initiation and may therefore improve overall survival [11]. In addition, in resource-limited settings, the focus is on more life-threatening opportunistic infections and skin conditions may be overlooked [12]. Finally, in many countries there is a lack of specialized dermatologists and the front line provision of HIV care is by primary care level nonspecialists that include medical officers, nurses, and midwives with minimal training in treatment of skin conditions [7, 13].

There is a need for clear practical but evidence-based guidance on the management of skin conditions in HIV-infected individuals. In this paper we review availability of national and professional society guidelines on HIV-related skin conditions and assess their accessibility, comprehensiveness, and the quality of evidence employed.

## 2. Methods

### 2.1. Search Strategy

We formulated a structured and comprehensive search strategy, using both peer-reviewed and gray literature as described in more detail below, to identify treatment guidelines for HIV-related skin conditions. Gray literature encompasses publications from governments, nongovernmental organizations, and societies that usually do not fulfil strict bibliographical requirements that are apparent in peer-reviewed literature [14]. The inclusion of gray literature sources was key in this process, since guidelines are not consistently or routinely included in peer-reviewed literature or in databases such as PubMed. Treatment guidelines were defined as a set of recommendations used to treat skin conditions in HIV-infected patients. Guidelines for children, adolescents, and adults were included as well as guidelines written in languages other than English.

### 2.2. Gray Literature Search

The gray literature search was performed in June 2014 and was divided into two categories: national guidelines and society guidelines. National guidelines were defined as guidelines developed by federal governments. From the UNAIDS database, 30 countries with highest HIV prevalence and 30 countries with the highest estimated number of people living with HIV were included [15]. Both lists were merged and duplicates were eliminated to yield a total of 43 countries. An additional 7 countries active in HIV/AIDS policy were selected on a discretionary basis to bring the list to a total of 50 countries (see the Appendix). The following specific databases along with web searches (Google, Google scholar) were used to obtain country-specific guidelines: World Health Organization (WHO), database of national HIV and TB guidelines, 2005–2011 [16]; USAID, AIDSTAR-One, National Treatment Guidelines [17]; the Interagency Task Team on the Prevention and Treatment of HIV Infection in Pregnant Women, Mothers and Children [18]; AIDSspace, Document Library [19]; and the Ministry of Health website for each country.

Society guidelines were defined as guidelines developed by nonprofit, nongovernmental organizations such as the Infectious Disease Society of America (IDSA) or international agencies such as the WHO (see the Appendix). Guidelines were also included from societies in dermatology, infectious diseases, and HIV/AIDS. These were obtained from the organizations' webpages.

### 2.3. Peer-Reviewed Literature Search

The peer-reviewed literature search was carried out on PubMed using the Cochrane HIV/AIDS Group's existing validated strategies to identify articles relevant to HIV infection and AIDS along with MeSH terms and relevant keywords to identify treatment guidelines for associated skin conditions [20].

### 2.4. Inclusion Criteria

For both gray and peer-reviewed literature, duplicate guidelines and older versions of the same guideline were excluded. Guidelines were included if they mentioned the treatment of one or more of ten selected HIV-related skin conditions: Kaposi's sarcoma, scabies, seborrheic dermatitis, molluscum contagiosum, eosinophilic folliculitis, papular pruritic eruption, varicella/herpes zoster, tinea, oropharyngeal candidiasis, and drug reactions (Stevens-Johnson syndrome or toxic epidermal necrolysis). Our search was restricted to ten skin conditions to allow us to perform an extensive gray literature search. These ten conditions are representative of other HIV-related skin conditions in terms of high disease burden, available evidence, effective interventions, and applicability in resource-limited settings.

### 2.5. Data Collection and Analysis of Guidelines

After screening and selection, we analyzed eligible guidelines with regard to the publication date, the frequency of specific skin conditions represented, the category of source document where the treatment for HIV-related skin conditions was mentioned, and the methodology that was used to develop guidelines. The type of guideline under which treatment guidelines for HIV-related skin conditions are found is an important factor to assess how quickly and easily a busy health care professional can access such treatment information. To address this issue of accessibility, we defined different types of guidelines as follows: HIV/AIDS treatment guidelines (guidelines that address treatment of HIV/AIDS using antiretroviral therapies), disease-specific treatment guidelines (treatment for one of the ten skin conditions associated with HIV such as scabies or tinea), STD/STI treatment guidelines (treatment of sexually transmitted infections (STDs)), skin disease treatment guidelines (treatment for skin diseases in general), opportunistic infections treatment guidelines (treatment for HIV-associated opportunistic infections), and standard clinical treatment guidelines (treatment for general medical conditions such as cardiovascular disorders and dermatological disorders).

We also assessed the methodology that was used to develop the treatment guidelines. We searched each document to determine which of the following methods were employed: expert opinion (based on experts' experience), scientific literature (based on results from clinical studies), graded evidence and strength of recommendations (based on rating systems such as Grading of Recommendations, Assessment, Development and Evaluation (GRADE) [21]), and adaptations from other guidelines (based on WHO and/or other guidelines).

### 2.6. Statistical Analysis

The countries were categorized by gross national income as defined by the World Bank: high income, middle income (upper middle and lower middle), and low income [22]. We tested the hypothesis that high-income countries would employ higher levels of evidence quality in their guideline development (Fisher's exact test).

## 3. Results

**Figure 1:**
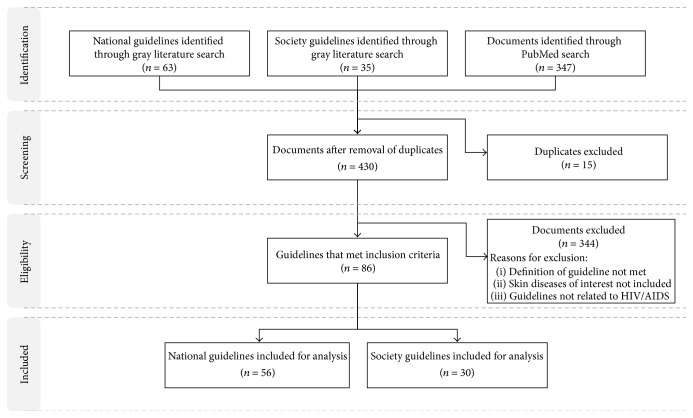
Prisma diagram showing selection process of treatment guidelines for HIV-related skin conditions.

As of June 2014, the gray and peer-reviewed literature search yielded a total of 430 potential guidelines once duplicates were removed, of which 86 guidelines (56 national and 30 society guidelines) met our selection criteria related to treatment guidelines for HIV-related skin conditions (Figure 1). The society guidelines were obtained from organizations like the WHO, American Academy of Dermatology (AAD), British HIV Association, IDSA, and others (see the Appendix). Of the fifty countries assessed for national guidelines (see the Appendix), fifteen did not have national guidelines for the treatment of HIV-related skin conditions although Australia and the United Kingdom had guidelines from societies. Included guidelines were in English, Ukrainian, Indonesian, Spanish, Portuguese, French, and Chinese.

Guidelines identified ranged from the years 1997 to 2014, with almost half (45%) more than five years old. Not all HIV-related skin conditions were included in each guideline. Among 86 total guidelines, oropharyngeal candidiasis, varicella/zoster, and Kaposi's sarcoma were most frequently addressed (62%, 60%, and 50%, resp.), whereas eosinophilic folliculitis, tinea, and papular pruritic eruption were the least represented (9%, 21%, and 26%, resp.).

### 3.1. Accessibility of Treatment Guidelines for HIV-Related Skin Conditions

**Figure 2:**
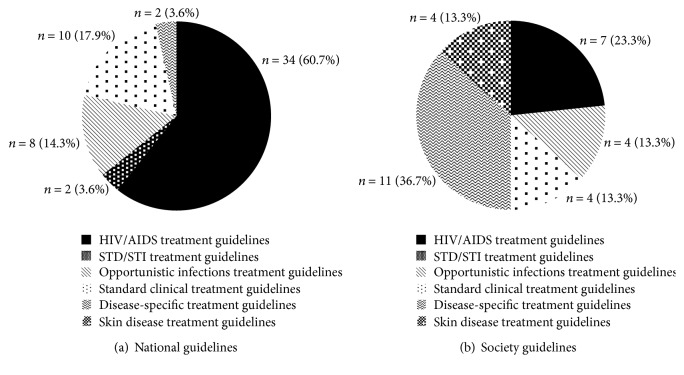
Categories of source documents where treatment of HIV-related skin conditions was mentioned.

Accessibility of the guidelines, defined as how easily treatment for HIV-related skin conditions are found, was poor. When searches of the gray literature were limited to guidelines that were specifically for HIV-related skin conditions, only two guidelines were identified, from AAD (1997) and the New York State Department of Health AIDS Institute (2004) [23, 24]. In the remaining 84 guidelines, treatment for different HIV-related skin conditions was mentioned within the context of HIV treatment in general (*n* = 41, 49%), STD guidelines (*n* = 2, 2%), skin disease treatment guidelines (*n* = 2, 2%), standard clinical treatment guidelines (*n* = 14, 17%), opportunistic infections treatment guidelines (*n* = 12, 14%), and disease-specific treatment guidelines (*n* = 13, 15%) (Table 1). Among national guidelines produced by specific countries' governments, the treatment guidelines for HIV-related skin conditions were found within five different types of guidelines, with the majority from HIV/AIDS treatment guidelines (61%) and none from skin disease treatment guidelines (Figure 2(a)). In contrast, among guidelines produced by societies, this information was most frequently contained within the disease-specific treatment guidelines (37%) (Figure 2(b)).

With respect to the ease of finding treatment guidelines for HIV-related skin conditions only 19 out of the 56 national guidelines had a dedicated dermatology section. For the remaining 37 national guidelines, treatment information for different HIV-related skin conditions was dispersed throughout the guideline.

### 3.2. Methodology Used to Develop Guidelines

**Figure 3:**
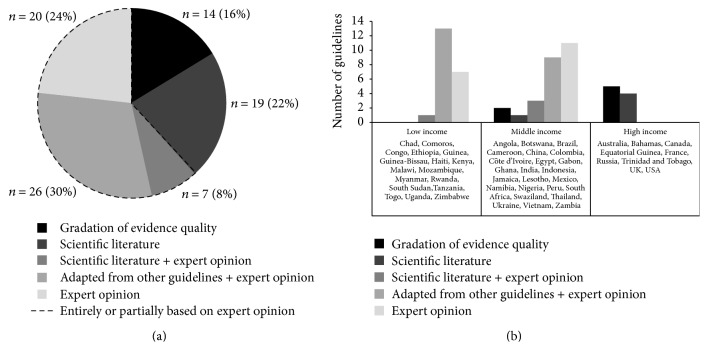
(a) Methodologies used to develop guidelines for HIV-related skin conditions (*n* = 86). (b) Association between gross national income of countries and the methodology used to develop guidelines for HIV-related skin conditions.

There was a wide variability in the methods used to develop the treatment guidelines for HIV-related skin conditions, with most relying on expert opinion either partially or completely (*n* = 53, 62%) rather than evidence-based scientific literature (Figure 3(a), Table 1). Guidelines frequently combined multiple methodologies such as adaptation from other guidelines along with expert opinion (30%) or scientific literature combined with expert opinion (8%). Only 14 guidelines (16%) employed the highest quality of guideline development process, which involves a rating system to grade the quality of evidence. The rating systems varied and included GRADE, Oxford Centre for Evidence-Based Medicine (CEBM), US Preventative Services Task Force (USPSTF), IDSA/US Public Health Service (IDSA/USPHS), or adaptations thereof.

Gross national income was correlated with the guideline methodology employed by national governments (Figure 3(b)). Low-income countries primarily adapted their guidelines from other sources (13 of 21) or relied on expert opinion (7 of 21). Middle-income countries relied on expert opinion (11 of 26) and adaptation from other guidelines (9 of 26), while a minor subset employed scientific literature (1 of 26) and gradation of evidence quality (2 of 26). High-income countries had either reviewed the scientific literature or used the gold standard of guideline development, gradation of evidence quality. High-income countries were significantly more likely to have employed an assessment of evidence quality in their guidelines (5 of 9 guidelines) as compared to lower- and middle-income countries (2 of 47 guidelines; *p* = 0.001).

## 4. Discussion

Our study suggests that there is a paucity of comprehensive evidence-based guidelines that is specific for treatment of HIV-related skin conditions. Currently, either HIV-related skin condition treatment information is difficult to find within the different types of guidelines or the methodology used to prepare the guidelines is not based on clinical evidence. Additionally, many guidelines are outdated and information across the prominent HIV-related skin conditions is fragmented across several guidelines. Together, these gaps highlight the need for an evidence-based, easily accessible summary guideline document for HIV-related skin conditions.

For a lay searcher or busy health professional, accessibility of current guidelines is poor. Firstly, to find treatment regimens for HIV-related skin conditions, entire guidelines written for other purposes such as HIV/AIDS treatment or opportunistic infections must be hand-searched. This process is time-consuming and represents a barrier to access by busy health care professionals. Furthermore, information for the major HIV-related skin conditions is scattered across several guidelines, requiring reference to multiple guidelines when trying to develop a treatment plan.

The guidelines identified rarely used evidence-based medicine. Methodology for guideline development varied widely, including a large subset based completely or partially on expert opinion (62%). Treatment decisions based on unvalidated information may be more harmful than helpful and may lead to increased morbidity and even mortality [21, 25]. Generally, treatment of skin conditions is often not evidence-based due to the lack of high quality studies and reliance on expert opinion is warranted under these circumstances. Further studies are needed to see whether recommendations differ between expert opinions and evidence-based medicine for treatment of skin conditions. In the smaller portion of guidelines that did employ gradation of evidence quality (16%), there were several grading systems, including GRADE, IDSA, CEBM, and USPSTF. High-income countries were much more likely to employ evidence-based medicine, in contrast to lower-income countries with higher HIV prevalence rates and arguably more in need of quality guidelines. In low-income countries, guidelines were primarily adapted from the WHO. This reliance on global guidance further highlights the need for high quality international guidelines.

Finally, almost half of the guidelines analyzed are more than five years old, which limits healthcare professionals to using dated information to address HIV-related skin conditions. Guidelines should be reassessed for validity and updated every 3 years for it to be useful to clinicians [26].

Our study has several limitations. With gray literature research, it is possible to miss guidelines due to the nature of gray literature records that might not be accessible through conventional searches. Our search was performed in June 2014 and guidelines that became available online since then could have been missed. Additionally, only documents which specified “HIV” or “AIDS” were included, whereas general immune deficiency or impairment was not considered. Guidelines written in local languages might also have been overlooked because they were not searchable in English. Finally, we restricted the review to the 10 highest burden HIV-related skin conditions and the selected 50 countries. We make the assumption that these parameters are representative of HIV-related skin condition treatment guidelines globally.

This review highlights the need for an effective guideline document for busy healthcare professionals to treat HIV-related skin conditions and identifies a gap in guidelines development within the field of dermatology. A comprehensive treatment guideline for HIV-related skin conditions should be a compilation of the most up-to-date treatment recommendations that are strictly vetted through a rating system for the evidence quality and strength of recommendations (such as GRADE) [21]. Recurrent updating would arm healthcare professionals with the latest treatment information. In response to these previously unmet needs, as identified in this work, the WHO has developed new set of guidelines for the treatment of the ten HIV-related skin conditions that were discussed in this study [27]. These guidelines were developed using the Cochrane systematic review and the GRADE rating system. Additionally, these guidelines address the local needs and constraints within resource-limited settings by considering factors such as availability of medications and costs of drugs. With the adoption of such guidelines, which can be accessed and adapted to different health systems, we can hope to see a decrease in morbidity and mortality related to HIV-related skin conditions.

## Figures and Tables

**Table 1:** Summary of national and society guidelines for the treatment of HIV-related skin conditions.

National guidelines (*n* = 56)		Type of guidelines							Methodology				

Country income category	List of countries	HIV/AIDS	STD	Opportunistic	Gen. clinical	Single disease	Skin disease		Gradation of quality	Scientific lit. (SL)	Expert opin. (EO)	SL + EO	Adapted + EO

Low income (17 countries)	Chad, Comoros, Congo, Ethiopia, Guinea, Guinea-Bissau, Haiti, Kenya, Malawi, Mozambique, Myanmar, Rwanda, South Sudan, Tanzania, Togo, Uganda, Zimbabwe	13	0	3	5	0	0		0	0	7	1	13
Middle income (24 countries)	Angola, Botswana, Brazil, Cameroon, China, Colombia, Côte d'Ivoire, Egypt, Gabon, Ghana, India, Indonesia, Jamaica, Lesotho, Mexico, Namibia, Nigeria, Peru, South Africa, Swaziland, Thailand, Ukraine, Vietnam, Zambia	18	0	2	5	0	0		2	1	11	3	9
High income (9 countries)	Australia, Bahamas, Canada, Equatorial Guinea, France, Russia, Trinidad and Tobago, UK, USA	3	2	3	0	2	0		5	4	0	0	0
Society guidelines (*n* = 30)		7	0	4	4	11	4		7	14	2	3	4

HIV/AIDS: HIV/AIDS treatment guidelines

STD: STD/STI treatment guidelines

Opportunistic: opportunistic infections treatment guidelines

Gen. clinical: standard clinical treatment guidelines

Single disease: disease-specific treatment guidelines

Skin disease: skin disease treatment guidelines

Gradation of quality: gradation of evidence quality

SL: scientific literature

EO: expert opinion

Adapted: adapted from other guidelines.

**Table 2:** National guidelines.

Country	Type of guideline	Year	Publishing body	Title	Disease treatment included	Methodology	Link
Angola	HIV/AIDS treatment guidelines	2011	Ministry of Health	Normas de Tratamento Anti-Retroviral	(i) Drug reactions	Adapted from other guidelines	http://www.emtct-iatt.org/wp-content/uploads/2013/04/Angola_National-ARV-Guidelines_2011.pdf

Australia	No national guidelines identified; however, guidelines exist from societies (see society guidelines)						

Bahamas	HIV/AIDS treatment guidelines	2005	Caribbean Epidemiology Centre	Caribbean Guidelines for the Care and Treatment of Persons with HIV Infection	(i) Oral candidiasis (ii) Seborrheic dermatitis (iii) Varicella/herpes zoster (iv) Molluscum contagiosum (v) Scabies (vi) Eosinophilic folliculitis (vii) Kaposi's sarcoma (viii) Drug reactions (ix) Papular pruritic eruptions	Scientific literature + expert opinion	http://www.who.int/hiv/pub/guidelines/caribbean_art.pdf

Botswana	HIV/AIDS treatment guidelines	2007	Ministry of Health	Acute Care: Botswana Integrated Management for HIV/AIDS and Other Illness	(i) Oral candidiasis (ii) Scabies (iii) Tinea (iv) Varicella/herpes zoster (v) Drug reactions (vi) Seborrheic dermatitis (vii) Molluscum contagiosum (viii) Papular pruritic eruption	Adapted from other guidelines	http://www.gov.bw/global/moh/pc_moh_07.pdf
	HIV/AIDS treatment guidelines	2012	Ministry of Health	Botswana National HIV & AIDS Treatment Guidelines	(i) Varicella/herpes zoster (ii) Oral candidiasis (iii) Kaposi's sarcoma (iv) Drug reactions	Expert opinion	http://www.med.upenn.edu/botswana/user_documents/BotsNatHIV-AIDSTreatGuideWEB22-05-2012.pdf

Brazil	HIV/AIDS treatment guidelines	2008	Ministry of Health	Recomendacoes para Terapia Antirretroviral em Adultos Infectados pelo HIV 2008	(i) Kaposi's sarcoma	CEBM	http://www.aidstar-one.com/sites/default/files/Brazil_National_Treatment_Guidelines_2008.pdf

Cameroon	HIV/AIDS treatment guidelines	2004	Ministry of Public Health	Guide National de Prise en Charge des Personnes Vivant avec le VIH/SIDA-Cameroun	(i) Oral candidiasis (ii) Kaposi's sarcoma (iii) Scabies (iv) Varicella/herpes zoster (v) Seborrheic dermatitis (vi) Molluscum contagiosum	Expert opinion	http://www.aidstar-one.com/sites/default/files/treatment/national_treatment_guidelines/Cameroon_tagged.pdf

Canada	STD/STI treatment guidelines	2008	Public Health Agency of Canada	Canadian Guidelines on Sexually Transmitted Diseases	(i) Scabies	USPSTF	http://www.phac-aspc.gc.ca/std-mts/sti-its/cgsti-ldcits/index-eng.php#toc

Chad	None identified						

China	None identified						

Colombia	HIV/AIDS treatment guidelines	2010	Ministry of Health and Social Protection	Guia para el Manejo de VIH/SIDA Basada en la Evidencia	(i) Drug reactions	IDSA-USPHS	http://www.aidsspace.org/upload_desc.php?user=7977&upid=2030

Comoros	HIV/AIDS treatment guidelines	2007	Ministry of Health	Guide de Prise en Charge de L'Infection a VIH aux Comoros	(i) Oral candidiasis (ii) Seborrheic dermatitis (iii) Scabies (iv) Drug reactions (v) Papular pruritic eruptions (vi) Varicella/herpes zoster (vii) Kaposi's sarcoma (viii) Molluscum contagiosum	Adapted from other guidelines + expert opinion	http://www.aidstar-one.com/sites/default/files/Comoros_2007_tagged_0.pdf

Congo	None identified						

Côte d'Ivoire	HIV/AIDS treatment guidelines	2005	Ministry of Health and Population	Guide de Prise en Charge de L'Infection a VIH/SIDA de L'Adulte et de L'Enfante	(i) Oral candidiasis (ii) Papular pruritic (iii) Drug reactions (iv) Varicella/herpes zoster (v) Seborrheic dermatitis (vi) Kaposi's sarcoma (vii) Molluscum contagiosum	Expert opinion	http://www.aidsspace.org/upload_desc.php?user=7977&upid=1921

Egypt	None identified						

Equatorial Guinea	None identified						

Ethiopia	Opportunistic infections treatment guidelines	2008	Federal Ministry of Health	Guidelines for Management of Opportunistic Infections and Antiretroviral Treatment in Adolescents and Adults in Ethiopia	(i) Varicella/herpes zoster (ii) Molluscum contagiosum (iii) Tinea (iv) Scabies (v) Oral candidiasis (vi) Drug reactions (vii) Papular pruritic eruption (viii) Kaposi's sarcoma	Adapted from other guidelines + expert opinion	http://www.who.int/hiv/pub/guidelines/ethiopia_art.pdf
	Standard treatment guidelines	2010	Drug Administration and Control Authority of Ethiopia	Standard Treatment Guideline for Primary Hospitals	(i) Varicella/herpes zoster	Expert opinion	http://apps.who.int/medicinedocs/documents/s17820en/s17820en.pdf

France	HIV/AIDS treatment guidelines	2010	Ministry of Health and Sports	Prise en Charge Médicale des Personnes Infectées par le VIH	(i) Kaposi's sarcoma (ii) Varicella/herpes zoster (iii) Oral candidiasis	CEBM	http://www.sante.gouv.fr/IMG/pdf/Rapport_2010_sur_la_prise_en_charge_medicale_des_personnes_infectees_par_le_VIH_sous_la_direction_du_Pr-_Patrick_Yeni.pdf

Gabon	None identified						

Ghana	Standard treatment guidelines	2010	Ministry of Health	Standard Treatment Guidelines	(i) Oral candidiasis (ii) Varicella/herpes zoster (iii) Seborrheic dermatitis	Expert opinion	http://apps.who.int/medicinedocs/documents/s18015en/s18015en.pdf

Guinea	HIV/AIDS treatment guidelines	2011	Ministry of Health and Hygiene	Normes et Protocoles de Prise en Charge de L'Infection par le VIH chez L'Adulte et L'Enfant en Guinee	(i) Oral candidiasis (ii) Varicella/herpes zoster (iii) Kaposi's sarcoma	Expert opinion	http://www.who.int/hiv/pub/guidelines/guinea_art.pdf?ua=1

Guinea-Bissau	None identified						

Haiti	HIV/AIDS treatment guidelines	2010	Ministry of Public Health and Population	Manuel de Normes de Prise en Charge Clinique et Therapeutique des Adolescents et Adultes Vivant avec le VIH/SIDA	(i) Oral candidiasis (ii) Varicella/herpes zoster (iii) Papular pruritic eruptions (iv) Seborrheic dermatitis (v) Kaposi's sarcoma	Expert opinion	http://www.aidsspace.org/upload_desc.php?user=7977&upid=1914

India	Opportunistic infections treatment guidelines	2007	National AIDS Control Organisation	Guidelines for Prevention and Management of Common Opportunistic Infections/Malignancies among HIV-Infected Adult and Adolescent	(i) Oral candidiasis (ii) Seborrheic dermatitis (iii) Varicella/herpes zoster (iv) Molluscum contagiosum (v) Scabies (vi) Kaposi's sarcoma (vii) Tinea	Expert opinion	http://naco.gov.in/upload/Policies%20&%20Guidelines/7-Guidelines%20for%20Prevention%20and%20Management%20of%20common%20opportunistic%20infections.pdf

Indonesia	HIV/AIDS treatment guidelines	2011	Ministry of Health	Tatalaksana Klinis Infeksi HIV dan Terapi Antiretroviral	(i) Varicella/herpes zoster (ii) Oral candidiasis (iii) Kaposi's sarcoma	Adapted from other guidelines + expert opinion	http://www.spiritia.or.id/Dok/pedomanart2011.pdf

Jamaica	HIV/AIDS treatment guidelines	2005	Caribbean Epidemiology Centre	Caribbean Guidelines for the Care and Treatment of Persons with HIV Infection	(i) Oral candidiasis (ii) Seborrheic dermatitis (iii) Varicella/herpes zoster (iv) Molluscum contagiosum (v) Scabies (vi) Eosinophilic folliculitis (vii) Kaposi's sarcoma (viii) Drug reactions (ix) Papular pruritic eruptions	Scientific literature + expert opinion	http://www.who.int/hiv/pub/guidelines/caribbean_art.pdf

Kenya	HIV/AIDS treatment guidelines	2005	Ministry of Health	Guidelines for Antiretroviral Drug Therapy in Kenya	(i) Drug reactions	Expert opinion	http://www.who.int/hiv/pub/guidelines/kenya_art.pdf
	Opportunistic infections treatment guidelines	2008	Ministry of Health	National Manual for the Management of HIV-Related Opportunistic Infections and Conditions	(i) Oral candidiasis (ii) Kaposi's sarcoma (iii) Scabies (iv) Papular pruritic eruptions (v) Tinea (vi) Varicella/herpes zoster (vii) Drug reactions (viii) Molluscum contagiosum (ix) Seborrheic dermatitis	Adapted from other guidelines + expert opinion	http://nascop.or.ke/library/ART%20guidelines/National%20Manual%20for%20the%20management%20of%20HIV%20related%20OIs.pdf
	Standard treatment guidelines	2009	Ministry of Medical Services	Clinical Guidelines for the Management and Referral of Common Conditions at Levels 2-3: Primary Care	(i) Oral candidiasis (ii) Seborrheic dermatitis (iii) Varicella/herpes zoster (iv) Kaposi's sarcoma	Adapted from other guidelines + expert opinion	http://apps.who.int/medicinedocs/documents/s20999en/s20999en.pdf

Lesotho	Standard treatment guidelines	2005	Ministry of Health and Social Welfare	Standard Treatment Guidelines and Essential Medicines List for Lesotho 2005	(i) Oral candidiasis (ii) Varicella/herpes zoster (iii) Tinea	Expert opinion	http://www.who.int/selection_medicines/country_lists/lso_2005_STGs_EML.pdf
	HIV/AIDS treatment guidelines	2007	Ministry of Health and Social Welfare	Working Draft-Lesotho National ART Guidelines	(i) Oral candidiasis (ii) Scabies (iii) Kaposi's sarcoma (iv) Varicella/herpes zoster (v) Molluscum contagiosum (vi) Tinea (vii) Seborrheic dermatitis (viii) Drug reactions	Expert opinion	http://www.who.int/hiv/pub/guidelines/lesotho_art.pdf

Malawi	HIV/AIDS treatment guidelines	2006	Ministry of Health	Treatment of AIDS: Guidelines for the Use of Antiretroviral Therapy in Malawi	(i) Kaposi's sarcoma	Expert opinion	https://www.google.com/url?sa=t&rct=j&q=&esrc=s&source=web&cd=1&cad=rja&uact=8&ved=0CB0QFjAA&url=http%3A%2F%2Fwww.hivunitmohmw.org%2Fuploads%2FMain%2FMalawi-ARV-Guidelines-2ndEdition.doc&ei=P8acU9WcAeXLsAThlYGwAw&usg=AFQjCNGw6k6cSW6YKODVanuFT9lM51jxag&sig2=sk9DWLIvD9yr8onBg6B5zg&bvm=bv.68911936,d.cWc
	Standard treatment guidelines	2009	Ministry of Health	Malawi Standard Treatment Guidelines 4th Edition 2009	(i) Kaposi's sarcoma (ii) Varicella/herpes zoster (iii) Drug reactions	Adapted from other guidelines + Expert opinion	http://apps.who.int/medicinedocs/documents/s18801en/s18801en.pdf
	Opportunistic infections treatment guidelines	2010	Ministry of Health	Guidelines for the Management of HIV-Related Illnesses in Paediatrics	(i) Oral candidiasis (ii) Tinea (iii) Molluscum contagiosum (iv) Kaposi's sarcoma (v) Papular pruritic eruptions (vi) Varicella/herpes zoster (vii) Scabies (viii) Drug reactions	Expert opinion	http://www.medcol.mw/paediatrics/uploads/HIVguidelines.pdf
	HIV/AIDS treatment guidelines	2011	Ministry of Health	Clinical Management of HIV in Children and Adults	(i) Oral candidiasis (ii) Kaposi's sarcoma (iii) Varicella/herpes zoster (iv) Seborrheic dermatitis (v) Tinea (vi) Papular pruritic eruptions	Adapted from other guidelines	http://www.who.int/hiv/pub/guidelines/malawi_art.pdf

Mexico	None identified						

Mozambique	HIV/AIDS treatment guidelines	2010	Ministry of Health	Guia de Tratamento Antiretroviral e Infeccoes Oportunistas no Adulto, Adolescente e Gravidas	(i) Oral candidiasis (ii) Varicella/herpes zoster (iii) Tinea (iv) Scabies (v) Seborrheic dermatitis (vi) Papular pruritic eruptions (vii) Kaposi's sarcoma (viii) Drug reactions	Expert opinion	http://www.who.int/hiv/pub/guidelines/mozambique_art.pdf?ua=1

Myanmar	HIV/AIDS treatment guidelines	2011	Ministry of Health	Guidelines for the Clinical Management of HIV Infection in Adults and Adolescents in Myanmar	(i) Varicella/herpes zoster (ii) Seborrheic dermatitis (iii) Papular pruritic eruptions (iv) Scabies (v) Oral candidiasis (vi) Kaposi's sarcoma	Adapted from other guidelines + expert opinion	http://aidstar-one.com/sites/default/files/Myanmar_2011.pdf

Namibia	Standard clinical treatment guidelines	2011	Ministry of Health and Social Services	Namibia Standard Treatment Guidelines	(i) Kaposi's sarcoma (ii) Scabies (iii) Seborrheic dermatitis (iv) Molluscum contagiosum (v) Eosinophilic folliculitis (vi) Drug reactions (vii) Varicella/herpes zoster (viii) Tinea (ix) Oral candidiasis	Expert opinion	http://apps.who.int/medicinedocs/documents/s19260en/s19260en.pdf
	HIV/AIDS treatment guidelines	2014	Ministry of Health and Social Services	National Guidelines for Antiretroviral Therapy	(i) Drug reactions	Adapted from other guidelines + expert opinion	http://preventcrypto.org/wp-content/uploads/2012/07/Namibia-National-ART-guidelines-2014.pdf

Nigeria	HIV/AIDS treatment guidelines	2007	Federal Ministry of Health	National Guidelines for HIV and AIDS Treatment and Care in Adolescents and Adults	(i) Oral candidiasis (ii) Varicella/herpes zoster (iii) Kaposi's sarcoma (iv) Scabies (v) Drugs reactions	Scientific literature + expert opinion	http://www.who.int/hiv/amds/Nigeria_adult_2007.pdf

Peru	None identified						

Russia	None identified						

Rwanda	HIV/AIDS treatment guidelines	2007	Ministry of Health	Guide de Prise en Charge des Personnes Infectées par le VIH au Rwanda	(i) Oral candidiasis (ii) Varicella/herpes zoster (iii) Molluscum contagiosum	Adapted from other guidelines + expert opinion	http://www.aidstar-one.com/sites/default/files/treatment/national_treatment_guidelines/Rwanda_2007_cleaner_version_tagged.pdf
	HIV/AIDS treatment guidelines	2013	Ministry of Health	National Guidelines for Prevention and Management of HIV, STIs & Other Blood Borne Infections	(i) Oral candidiasis (ii) Varicella/herpes zoster (iii) Kaposi's sarcoma	Adapted from other guidelines + expert opinion	http://www.aidsspace.org/upload_desc.php?user=7977&upid=2149

South Africa	HIV/AIDS treatment guidelines	2010	National Department of Health	The South African Antiretroviral Treatment Guidelines 2010	(i) Oral candidiasis (ii) Varicella/herpes zoster (iii) Seborrheic dermatitis (iv) Molluscum contagiosum (v) Tinea (vi) Drug reactions (vii) Kaposi's sarcoma	Expert opinion	http://www.aidstar-one.com/sites/default/files/South_Africa_National_HIV_Treatment_Guidelines_Combined_2010.pdf
	Standard clinical treatment guidelines	2012	National Department of Health	Standard Treatment Guidelines and Essential Drugs List for South Africa	(i) Drug reactions (ii) Oral candidiasis (iii) Kaposi's sarcoma	Scientific literature + expert opinion	http://www.health.gov.za/docs/Policies/2012/Standard_treatment_guidelines_and_essential_medicines_list_2012.pdf

South Sudan	None identified						

Swaziland	HIV/AIDS treatment guidelines	2010	Ministry of Health and Social Welfare	Swaziland Paediatric HIV/AIDS Treatment Guidelines	(i) Papular pruritic eruptions (ii) Molluscum contagiosum (iii) Varicella/herpes zoster (iv) Oral candidiasis (v) Kaposi's sarcoma (vi) Drug reactions	Adapted from other guidelines + expert opinion	http://www.emtct-iatt.org/wp-content/uploads/2013/04/Swaziland_National-Pediatric-HIV-Guidelines_2010.pdf
	HIV/AIDS treatment guidelines	2010	Ministry of Health and Social Welfare	National Comprehensive HIV Package of Care	(i) Drug reactions	Adapted from other guidelines + expert opinion	http://www.who.int/hiv/pub/guidelines/swaziland_art.pdf

Tanzania	HIV/AIDS treatment guidelines	2012	Ministry of Health and Social Welfare	National Guidelines for the Management of HIV and AIDS	(i) Oral candidiasis (ii) Papular pruritic eruptions (iii) Scabies (iv) Seborrheic dermatitis (v) Molluscum contagiosum (vi) Kaposi's sarcoma (vii) Varicella/herpes zoster	Adapted from other guidelines + expert opinion	http://pmtct.or.tz/wp-content/uploads/2013/03/ART-guidelines_PDF.pdf

Thailand	None identified						

Togo	None identified						

Trinidad and Tobago	HIV/AIDS treatment guidelines	2005	Caribbean Epidemiology Centre	Caribbean Guidelines for the Care and Treatment of Persons with HIV Infection	(i) Oral candidiasis (ii) Seborrheic dermatitis (iii) Varicella/herpes zoster (iv) Molluscum contagiosum (v) Scabies (vi) Eosinophilic folliculitis (vii) Kaposi's sarcoma (viii) Drug reactions (ix) Papular pruritic eruptions	Scientific literature + expert opinion	http://www.who.int/hiv/pub/guidelines/caribbean_art.pdf

Uganda	HIV/AIDS treatment guidelines	2009	Ministry of Health	National Antiretroviral Treatment Guidelines for Adults, Adolescents, and Children	(i) Drug reactions	Adapted from other guidelines + expert opinion	http://www.aidstar-one.com/sites/default/files/treatment/national_treatment_guidelines/Uganda_guidelines_2009.pdf

Uganda	Standard clinical treatment guidelines	2012	Ministry of Health	Uganda Clinical Guidelines 2012	(i) Drug reactions	Adapted from other guidelines + expert opinion	http://sure.ug/?download=UCG%202012.pdf

UK	No national guidelines identified; however, guidelines exist from societies (see society guidelines)						

Ukraine	Opportunistic infections treatment guidelines	2007	Ministry of Health	Clinical Protocol for the Treatment of Opportunistic Infections in Patients with HIV and AIDS	(i) Oral candidiasis (ii) Varicella/herpes zoster (iii) Kaposi's sarcoma	Adapted from other guidelines + expert opinion	http://moz.gov.ua/ua/portal/dn_20031212_580.html

USA	Opportunistic infections treatment guidelines	2004	Centers for Disease Control and Prevention	Treating Opportunistic Infections Among HIV-Infected Adults and Adolescents	(i) Oral candidiasis (ii) Varicella/herpes zoster	IDSA-USPHS	http://www.cdc.gov/mmwr/pdf/rr/rr5315.pdf
	STD/STI treatment guidelines	2010		Sexually Transmitted Diseases Treatment Guidelines, 2010	(i) Scabies	Scientific literature	http://www.cdc.gov/mmwr/preview/mmwrhtml/rr5912a1.htm
	Opportunistic infections treatment guidelines	2013		Guidelines for the Prevention and Treatment of Opportunistic Infections in HIV-Exposed and HIV-Infected Children	(i) Oral candidiasis (ii) Varicella/herpes zoster	IDSA-USPHS	http://aidsinfo.nih.gov/contentfiles/lvguidelines/oi_guidelines_pediatrics.pdf
	HIV/AIDS treatment guidelines	2011	US Department of Veteran Affairs	Dermatologic Conditions-Primary Care of Veterans with HIV	(i) Seborrheic dermatitis (ii) Eosinophilic folliculitis	Scientific literature	http://www.hiv.va.gov/provider/manual-primary-care/dermatologic.asp
	Disease-specific treatment guidelines	2011	Department of Health and Human Services	Clinical Information: Molluscum Contagiosum	(i) Molluscum contagiosum	Scientific literature	http://www.cdc.gov/ncidod/dvrd/molluscum/clinical_overview.htm
	Disease-specific treatment guidelines	2013	National Cancer Institute	Kaposi's Sarcoma Treatment	(i) Kaposi's sarcoma	Scientific literature	http://www.cancer.gov/cancertopics/pdq/treatment/kaposis/HealthProfessional/page3
	Opportunistic infections treatment guidelines	2013	Department of Health and Human Services	Guidelines for the Prevention and Treatment of Opportunistic Infections in HIV-Infected Adults and Adolescents	(i) Varicella/herpes zoster (ii) Oral candidiasis (iii) Kaposi's sarcoma	IDSA-USPHS	http://aidsinfo.nih.gov/guidelines/html/4/adult-and-adolescent-oi-prevention-and-treatment-guidelines/0

Vietnam	HIV/AIDS treatment guidelines	2009	Ministry of Health	Guidelines for HIV/AIDS Diagnosis and Treatment	(i) Oral candidiasis (ii) Varicella/herpes zoster (iii) Molluscum contagiosum (iv) Drug reactions	Adapted from other guidelines	http://www.aidsspace.org/upload_desc.php?user=7977&upid=2000

Zambia	HIV/AIDS treatment guidelines	2002	Central Board of Health	Integrated Technical Guidelines for Frontline Healthworkers	(i) Seborrheic dermatitis (ii) Papular pruritic eruptions (iii) Molluscum contagiosum	Expert opinion	http://pdf.usaid.gov/pdf_docs/PNADD029.pdf
	HIV/AIDS treatment guidelines	2008		Zambia HIV National Guidelines	(i) Oral candidiasis (ii) Varicella/herpes zoster (iii) Kaposi's sarcoma (iv) Seborrheic dermatitis (v) Molluscum contagiosum (vi) Eosinophilic folliculitis	Scientific literature	http://www.zambiahivguide.org/
	Standard clinical treatment guidelines	2008	Ministry of Health	Standard Treatment Guidelines, Essential Medicines List & Essential Laboratory Supplies List for Zambia	(i) Kaposi's sarcoma	Expert opinion	http://apps.who.int/medicinedocs/documents/s19280en/s19280en.pdf
	HIV/AIDS treatment guidelines	2010	Ministry of Health	Adult and Adolescent Antiretroviral Therapy Protocols 2010	(i) Drug reactions	Adapted from other guidelines + expert opinion	http://www.who.int/hiv/pub/guidelines/zambia_art.pdf?ua=1

Zimbabwe	Standard clinical treatment guidelines	2011	Ministry of Health and Child Welfare	6th Essential Drugs List and Standard Treatment Guidelines for Zimbabwe	(i) Varicella/herpes zoster (ii) Seborrheic dermatitis (iii) Drug reactions (iv) Kaposi's sarcoma (v) Papular pruritic eruptions (vi) Oral candidiasis	Scientific literature + expert opinion	http://globalhealth.stanford.edu/documents/EDLIZ%202011%206th%20edition.doc
	HIV/AIDS treatment guidelines	2013	Ministry of Health and Child Care	Guidelines for Antiretroviral Therapy for the Prevention and Treatment of HIV in Zimbabwe	(i) Drug reactions (ii) Kaposi's sarcoma	Adapted from other guidelines + expert opinion	https://www.google.com/url?sa=t&rct=j&q=&esrc=s&source=web&cd=1&cad=rja&uact=8&ved=0CCcQFjAA&url=http%3A%2F%2Fwww.emtct-iatt.org%2Fwp-content%2Fuploads%2F2014%2F05%2F2013-zimbabwe-arv-guidelines-main-document.pdf&ei=YPeMU_z2FqWmsASEjYHoBQ&usg=AFQjCNHchIkdbhZ14yZIM7SOV32vdCgnIQ&sig2=ZeVAun9vbaQxa2zXyVSaOA&bvm=bv.68191837,d.cWc

**Table 3:** Society guidelines.

Society	Type of guideline	Year	Title	Disease treatment included	Methodology	Link
AIDS MEDS	HIV/AIDS treatment guidelines	2011	Opportunistic Infections	(i) Kaposi's sarcoma (ii) Varicella/herpes zoster (iii) Molluscum contagiosum (iv) Oral candidiasis	Expert opinion	http://www.aidsmeds.com/articles/OIs_4898.shtml

American Academy of Dermatology (AAD)	Skin disease treatment guidelines	1997	Guidelines of Care for Dermatologic Conditions in Patients Infected with HIV	(i) Oral candidiasis (ii) Papular pruritic eruptions (iii) Kaposi's sarcoma (iv) Varicella/herpes zoster (v) Molluscum contagiosum (vi) Scabies (vii) Seborrheic dermatitis (viii) Eosinophilic folliculitis	Scientific literature	http://www-ncbi-nlm-nih-gov.ezp-prod1.hul.harvard.edu/pubmed/?term=Guidelines+of+care+for+dermatologic+conditions+in+patients+infected+with+HIV

American Cancer Society	Disease-specific treatment guidelines	2013	Kaposi Sarcoma	(i) Kaposi's sarcoma	Scientific literature	http://www.cancer.org/acs/groups/cid/documents/webcontent/003106-pdf.pdf

Australasian Society for HIV Medicine	Opportunistic infections treatment guidelines	2009	HIV Management in Australasia	(i) Scabies (ii) Seborrheic dermatitis (iii) Drug reactions (iv) Eosinophilic folliculitis (v) Papular pruritic eruptions (vi) Tinea (vii) Molluscum contagiosum (viii) Oral candidiasis (ix) Varicella/herpes zoster (x) Kaposi's sarcoma	Scientific literature + expert opinion	http://www.ashm.org.au/images/Publications/Monographs/HIV_Management_Australasia/HIV-Management-Australia-2009.pdf

British Association of Sexual Health and HIV	Disease-specific treatment guidelines	2008	United Kingdom National Guideline on the Management of Scabies Infestation	(i) Scabies	Gradation of evidence quality	http://www.bashh.org/documents/27/27.pdf
	Disease-specific treatment guidelines	2008	United Kingdom National Guideline on the Management of Molluscum contagiosum	(i) Molluscum contagiosum	Gradation of evidence quality	http://www.bashh.org/documents/26/26.pdf

British Columbia Centre for Excellence in HIV/AIDS	Opportunistic infections treatment guidelines	2009	Therapeutic Guidelines for Opportunistic Infections	(i) Oral candidiasis (ii) Varicella/herpes zoster	Scientific literature	http://www.cfenet.ubc.ca/sites/default/files/uploads/docs/Opportunistic_Infection_Therapeutic_Guidelines2009.pdf

British HIV Association (BHIVA)	Opportunistic infections treatment guidelines	2011	British HIV Association and British Infection Association Guidelines for the Treatment of Opportunistic Infection in HIV-Seropositive Individuals 2011	(i) Varicella/herpes zoster (ii) Scabies (iii) Oral candidiasis	CEBM	http://www.bhiva.org/documents/Guidelines/OI/hiv_v12_is2_Iss2Press_Text.pdf
	HIV/AIDS treatment guidelines	2014	British HIV Association Guidelines for HIV-Associated malignancies 2014	(i) Kaposi's sarcoma	GRADE	http://www.bhiva.org/documents/Guidelines/Malignancy/2014/MalignancyGuidelines2014.pdf

British Society for Sexual Health and HIV	Disease-specific treatment guidelines	2014	DRAFT-UK National Guideline for the Management of Genital Molluscum in Adults, 2014	(i) Molluscum contagiosum	CEBM	https://www.google.com/url?sa=t&rct=j&q=&esrc=s&source=web&cd=1&cad=rja&uact=8&ved=0CB0QFjAA&url=http%3A%2F%2Fwww.bashh.org%2Fdocuments%2FMC%2520draft%25202014.doc&ei=CoibU_–HMrmsASmxAE&usg=AFQjCNF1Dx4UQrTIYl-L3gbXqmQIZVbjkQ&sig2=2dBFgaS0PlOvgqjAMCW8_A&bvm=bv.68911936,d.cWc

Cancer Care Ontario	Disease-specific treatment guidelines	2013	Liposomal Anthracyclines in the Management of Patients with HIV-Positive Kaposi's Sarcoma: Guideline Recommendations	(i) Kaposi's sarcoma	Scientific literature	https://www.cancercare.on.ca/common/pages/UserFile.aspx?serverId=6&path=/File%20Database/CCO%20Files/PEBC/pebc12-8f.pdf

EACS European AIDS Clinical Society	HIV/AIDS treatment guidelines	2013	EACS European AIDS Clinical Society Guidelines	(i) Varicella/herpes zoster (ii) Oral candidiasis	Scientific literature	http://www.eacsociety.org/Portals/0/Guidelines_Online_131014.pdf

European Society of Clinical Microbiology and Infectious Diseases	Disease-specific treatment guidelines	2012	ESCMID Guideline for the Diagnosis and Management of Candida Diseases 2012: Patients with HIV Infection or AIDS	(i) Oral candidiasis	GRADE	https://www.escmid.org/fileadmin/src/media/PDFs/4ESCMID_Library/2Medical_Guidelines/ESCMID_Guidelines/ESCMID_Candida_Guidelines_CMI_Dec2012_HIV_AIDS.pdf

Family Health International 360	HIV/AIDS treatment guidelines	2004	HIV/AIDS Care and Treatment	(i) Scabies (ii) Varicella/herpes zoster (iii) Oral candidiasis (iv) Tinea (v) Molluscum contagiosum (vi) Papular pruritic eruptions (vii) Drug reactions (viii) Seborrheic dermatitis (ix) Kaposi's sarcoma	Scientific literature	http://www.fhi360.org/sites/default/files/media/documents/HIV-AIDS%20Care%20and%20Treatment%20Burundi%202004.pdf

German and Austrian AIDS Society	Opportunistic infections treatment guidelines	2013	Therapy and Prophylaxis of Opportunistic Infections in HIV-Infected Patients: A Guideline by the German and Austrian AIDS Societies	(i) Varicella/herpes zoster (ii) Oral candidiasis	Scientific literature + expert opinion	http://www.daignet.de/site-content/hiv-therapie/leitlinien-1/2013_Infection_OI_LL%20englisch%20neu.pdf

HIV Clinical Resource	Skin disease treatment guidelines	2004	Dermatologic Manifestations	(i) Oral candidiasis (ii) Tinea (iii) Varicella/herpes zoster (iv) Molluscum contagiosum (v) Scabies (vi) Seborrheic dermatitis (vii) Drug reactions	Scientific literature	http://www.hivguidelines.org/clinical-guidelines/infants-children/dermatologic-manifestations/

Infectious Diseases Society of America	Disease-specific treatment guidelines	2000	Practice Guidelines for the Treatment of Candidiasis. Infectious Diseases Society of America	(i) Oral candidiasis	Scientific literature	http://www-ncbi-nlm-nih-gov.ezp-prod1.hul.harvard.edu/pubmed/10770728
	Skin disease treatment guidelines	2011	Practice Guidelines for the Diagnosis and Management of Skin and Soft-Tissue Infections	(i) Varicella/herpes zoster	IDSA-USPHS	http://www.idsociety.org/uploadedFiles/IDSA/Guidelines-Patient_Care/PDF_Library/Skin%20and%20Soft%20Tissue.pdf

International Antiviral Society USA	Skin disease treatment guidelines	2006	Dermatologic Manifestations of HIV Infection	(i) Papular pruritic eruptions (ii) Molluscum contagiosum (iii) Drug reactions (iv) Kaposi's sarcoma	Expert opinion	https://www.iasusa.org/sites/default/files/tam/13-5-149.pdf

International Foundation for Dermatology (IFD)	Disease-specific treatment guidelines	?	Protocols/Management of Tinea Capitis	(i) Tinea	Scientific literature	http://www.ifd.org/protocols/tinea-capitis

International Foundation for Dermatology (IFD)	Disease-specific treatment guidelines	?	Reports & Management Protocols: Management of Scabies	(i) Scabies	Scientific literature	http://www.ifd.org/protocols/scabies

International Union against STIs (IUSTI)	Disease-specific treatment guidelines	2010	European Guideline for the Management of Scabies	(i) Scabies	Adapted from other guidelines	http://www.iusti.org/regions/Europe/pdf/2010/Euro_Guideline_Scabies_2010.pdf

Médecins Sans Frontiéres	Standard clinical treatment guidelines	2013	Clinical Guidelines: Diagnosis and Treatment Manual for Curative Programmes in Hospitals and Dispensaries	(i) Varicella/herpes zoster (ii) Scabies (iii) Oral candidiasis (iv) Seborrheic dermatitis (v) Tinea	Adapted from other guidelines + expert opinion	http://refbooks.msf.org/msf_docs/en/clinical_guide/cg_en.pdf

Stanley Ho Centre for Emerging Infectious Diseases	HIV/AIDS treatment guidelines	2007	HIV Manual 2007	(i) Varicella/herpes zoster (ii) Molluscum contagiosum (iii) Oral candidiasis (iv) Papular pruritic eruptions (v) Eosinophilic folliculitis	Scientific literature	http://www.info.gov.hk/aids/pdf/g190htm/iv_index.htm

The Cochrane Library	Disease-specific treatment guidelines	2010	Interventions for the Prevention and Management of Oropharyngeal Candidiasis Associated with HIV Infection in Adults and Children	(i) Oral candidiasis	Scientific literature	http://onlinelibrary.wiley.com/doi/10.1002/14651858.CD003940.pub3/abstract

WHO	HIV/AIDS treatment guidelines	2008	Integrated Management of Childhood Illness for High HIV Settings	(i) Oral candidiasis (ii) Seborrheic dermatitis (iii) Scabies (iv) Drug reactions (v) Papular pruritic eruptions (vi) Varicella/herpes zoster (vii) Tinea (viii) Molluscum contagiosum	Scientific literature + expert opinion	http://www-ncbi-nlm-nih-gov.ezp-prod1.hul.harvard.edu/pubmed/23805440
	Standard clinical treatment guidelines	2011	IMAI District Clinician Manual: Hospital Care for Adolescents and Adults	(i) Eosinophilic folliculitis (ii) Papular pruritic eruption (iii) Scabies (iv) Molluscum contagiosum (v) Varicella/herpes zoster (vi) Kaposi sarcoma	Adapted from other guidelines	http://www.who.int/influenza/patient_care/DCM_Volume_1.pdf
	HIV/AIDS treatment guidelines	2011	Manual on Paediatric HIV Care and Treatment for District Hospitals	(i) Oral candidiasis	Adapted from other guidelines + expert opinion	http://whqlibdoc.who.int/publications/2011/9789241501026_eng.pdf
	Standard clinical treatment guidelines	2014	Integrated Management of Childhood Illness	(i) Papular pruritic eruptions (ii) Tinea (iii) Scabies (iv) Varicella/herpes zoster (v) Seborrheic dermatitis (vi) Drug reactions	Scientific literature	http://apps.who.int/iris/bitstream/10665/104772/16/9789241506823_Chartbook_eng.pdf?ua=1
	Standard clinical treatment guidelines	2013	Pocket Book of Hospital Care for Children	(i) Oral candidiasis	Scientific literature	http://apps.who.int/iris/bitstream/10665/81170/1/9789241548373_eng.pdf?ua=1

## Appendix

See Tables 2 and 3.

## Abbreviations

AAD: American Academy of Dermatology
AIDS: Acquired Immunodeficiency Syndrome
cART: Combination antiretroviral therapy
CEBM: Centre for Evidence-Based Medicine
COPD: Chronic obstructive pulmonary disease
GRADE: Grading of Recommendations, Assessment, Development and Evaluation
HIV: Human Immunodeficiency Virus
IDSA: Infectious Disease Society of America
STDs: Sexually transmitted infections
TB: Tuberculosis
USPHS: US Public Health Service
USPSTF: US Preventative Services Task Force
WHO: World Health Organization.
